# Identification of cancer chemopreventive isothiocyanates as direct inhibitors of the arylamine *N*-acetyltransferase-dependent acetylation and bioactivation of aromatic amine carcinogens

**DOI:** 10.18632/oncotarget.7086

**Published:** 2016-01-30

**Authors:** Romain Duval, Ximing Xu, Linh-Chi Bui, Cécile Mathieu, Emile Petit, Kevin Cariou, Robert H. Dodd, Jean-Marie Dupret, Fernando Rodrigues-Lima

**Affiliations:** ^1^ Université Paris Diderot, Sorbonne Paris Cité, Unité BFA, CNRS UMR 8251, 75013 Paris, France; ^2^ Institut de Chimie des Substances Naturelles, CNRS UPR 2301, Université Paris-Saclay, 91198 Gif-sur-Yvette, France; ^3^ UFR Sciences du Vivant, Université Paris Diderot, 75013 Paris, France

**Keywords:** cancer chemoprevention, arylamine carcinogens, isothiocyanate phytochemicals, carcinogen metabolism, enzyme inhibition

## Abstract

Aromatic amines (AAs) are chemicals of industrial, pharmacological and environmental relevance. Certain AAs, such as 4-aminobiphenyl (4-ABP), are human carcinogens that require enzymatic metabolic activation to reactive chemicals to form genotoxic DNA adducts. Arylamine N-acetyltransferases (NAT) are xenobiotic metabolizing enzymes (XME) that play a major role in this carcinogenic bioactivation process. Isothiocyanates (ITCs), including benzyl-ITC (BITC) and phenethyl-ITC (PEITC), are phytochemicals known to have chemopreventive activity against several aromatic carcinogens. In particular, ITCs have been shown to modify the bioactivation and subsequent mutagenicity of carcinogenic AA chemicals such as 4-ABP. However, the molecular and biochemical mechanisms by which these phytochemicals may modulate AA carcinogens bioactivation and AA-DNA damage remains poorly understood.

This manuscript provides evidence indicating that ITCs can decrease the metabolic activation of carcinogenic AAs *via* the irreversible inhibition of NAT enzymes and subsequent alteration of the acetylation of AAs. We demonstrate that BITC and PEITC react with NAT1 and inhibit readily its acetyltransferase activity (k_i_ = 200 M^−1^.s^−1^ and 66 M^−1^.s^−1^ for BITC and PEITC, respectively). Chemical labeling, docking approaches and substrate protection assays indicated that inhibition of the acetylation of AAs by NAT1 was due to the chemical modification of the enzyme active site cysteine. Moreover, analyses of AAs acetylation and DNA adducts in cells showed that BITC was able to modulate the endogenous acetylation and bioactivation of 4-ABP. In conclusion, we show that direct inhibition of NAT enzymes may be an important mechanism by which ITCs exert their chemopreventive activity towards AA chemicals.

## INTRODUCTION

Aromatic amines (AAs) represent one of the most important class of industrial and environmental chemicals [1, 2]. AAs are used as raw materials or as intermediates in the manufacturing of drugs or industrial chemicals such as pesticides, dyes, polymers or rubbers. AAs are also byproducts of gasoline combustion and pyrolysis reactions [2]. Many AAs, such as the tobacco smoke component 4-aminobiphenyl (4-ABP), are known human carcinogens. Exposure to these aromatic chemicals is a causal factor in a multi-step process leading to the development of tumors [3, 4]. Like most chemical carcinogens, AAs require metabolic activation by xenobiotic-metabolizing enzymes (XME) in order to exert their carcinogenicity, in particular through reactive metabolites that interact covalently with DNA resulting in genotoxic adducts [1, 4]. Recent data also suggest that other mechanisms, unrelated to DNA-adduct formation, such as oxidative stress may also be of importance for AA-dependent carcinogenesis [5].

In addition to cytochromes P450 (CYP450), arylamine *N*-acetyltransferases (NATs), such as human NAT1 and NAT2, are phase II XME that play a major role in the detoxification and/or the bioactivation of AA carcinogens [4, 6–8]. NATs are polymorphic XME and several studies have revealed an association between NAT activities and the risk of developing environmentally-related cancers [4, 6, 7]. Cellular metabolic activation of procarcinogenic AAs relies on their *N*-hydroxylation by CYP450 (such as CYP1A1, 1A2 or 2E1), and further activation by NAT-catalyzed *O*-acetylation. This step results in acetoxy ester metabolites that spontaneously degrade to form arylnitrenium ions that can form bulky adducts on DNA leading to the production of mutations [1, 4]. It has been shown that 4-ABP DNA adducts and mutation levels were significantly higher in cells displaying elevated NAT1 activity which emphasizes the relative importance of NAT1-catalyzed *O*-acetylation of *N*-hydroxy-4-ABP in cancer risk [9]. Higher NAT2 catalytic activity was also found to be associated with higher 4-ABP-mediated cytotoxicity and DNA adduct formation [10].

The NAT-dependent bioactivation pathway of AAs is in competition with the NAT-mediated *N*-acetylation pathway which forms innocuous metabolites [4]. However, it has been shown that *N*-acetylation of certain AAs, such as benzidine, may nonetheless enhance metabolic activation [11].

Isothiocyanates (ITCs) are phytochemicals with potential cancer chemopreventive activity which are found in cruciferous vegetables [12]. Several ITCs, such as benzyl-ITC (BITC), phenethyl-ITC (PEITC) and sulforaphane (SFN) have demonstrated cancer preventive activity in animals and increased dietary intake of ITCs have been shown to be associated with a reduced cancer risk in humans [12, 13]. The electrophilic carbon residue in the ITC moiety reacts with biological nucleophiles such as cysteines in proteins or in glutathione. Modification of proteins is recognized as a key mechanism underlying the biological activity of ITCs [12–14]. The cancer chemopreventive activity of ITCs is attributed, at least in part, to their ability to alter the metabolic activation of procarcinogens through inhibition of CYP450 enzymes and nuclear factor E2-related factor (Nrf2)-dependent induction of phase II XME, such as glutathione S-transferase (GST) enzymes [12–14].

ITCs have been shown to alter the metabolic activation and subsequent mutagenicity and genotoxicity of AA carcinogens such as 4-ABP [15, 16]. However, the molecular and biochemical mechanisms by which these chemopreventive phytochemicals modulate AA carcinogen bioactivation and DNA damage remains poorly documented.

We report here molecular, kinetic and cellular evidence showing that ITCs, such as BITC, can modulate the bioactivation of carcinogenic AAs through irreversible inhibition of NAT enzymes. Alteration of the NAT-dependent metabolism of AAs by ITCs may represent a novel biochemical mechanism by which these phytochemicals exert their chemoprotective effects towards AA carcinogens.

## RESULTS AND DISCUSSION

**Figure 1:**
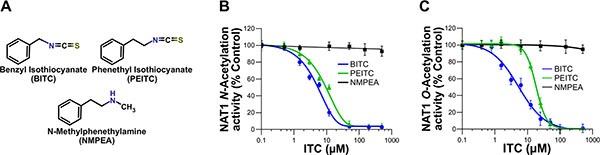
Dose-dependent inhibition of NAT1 activity by the aralkyl isothiocyanates benzyl isothiocyanate (BITC) and phenetyl isothiocyanate (PEITC) (**A**) Chemical structures of the ITCs (BITC and PEITC) and *N*-methylphenethylamine (NMPEA), a non-ITC analog of PEITC. (**B** and **C**) Effect of BITC, PEITC and NMPEA on NAT1 activity. NAT1 enzyme (1 μM) was incubated with increasing concentrations of BITC, PEITC and NMEPA for 30 min at 37°C. Residual NAT1 *N*-acetylation (B) and O-acetylation activities (C) were measured using *p*-aminobenzoic acid (pABA) and *N*-hydroxy-4-aminobiphenyl (*N*-OH-4-ABP) as AA substrates, respectively. Error bars indicate S.D. values. Results are presented as percent control NAT1 activity.

Phytochemical ITCs have been shown to exert chemopreventive properties towards certain carcinogenic AAs, such as 4-ABP [15, 16]. However, the molecular and cellular bases of this chemoprevention remain limited. Given the key role of NATs in the metabolic activation of AA carcinogens, we hypothesized that these enzymes could be targeted by chemopreventive ITCs such as BITC or PEITC (Figure 1A). In addition, it has been shown that increased activity of human NAT enzymes, in particular NAT1, is associated with higher AA-DNA adducts and mutagenesis which are relevant points for cancer risk [9, 10]. To address whether human NAT1 is a target of chemopreventive ITCs, purified recombinant enzyme was exposed to different concentrations of two most studied ITCs (BITC or PEITC) for a short period of time (30 min) [12–14]. As shown in Figure 1B and 1C, these two chemoprotective ITCs were found to inhibit the *N*- and *O*-arylamine acetyltransferase activities of NAT1 in a dose-dependent manner. Full inhibition of NAT1 activities were obtained with BITC and PEITC concentrations close to 30 μM. The IC_50_ values were 7 μM (± 2.5) and 15 μM (± 4.9) for BITC and PEITC, respectively. In contrast, NMPEA, a structural analog of PEITC without the ITC functionality, did not inhibit NAT1 activities. Sulforaphane (SFN), a dietary-ITC with an alkyl side chain was also found to inhibit NAT1 but at concentrations close to 100 μM (data not shown). These results are in agreement with previous studies on molecular targets of phytochemical ITCs [17–19]. For instance, it has been reported that the ITC functional group is essential for the inhibition of the proteasome activity by ITC phytochemicals, and that the side chains of ITCs dictates the inhibitory potency [17–19]. Moreover, the structural similarities of the arylalkyl side chains of BITC and PEITC with arylamine substrates of NATs may also contribute to the inhibitory potency of these ITCs towards NAT1 when compared to SFN. In addition to the experiments carried out with NAT1, analyses conducted with purified human NAT2 enzyme gave similar results with full inhibition of the enzyme by BITC and PEITC obtained at concentrations close to 25 μM (Supplementary Figure 1). Our data thus indicate that the enzymatic acetylation of AAs by NATs is readily inhibited *in vitro* by BITC and PEITC.

**Figure 2:**
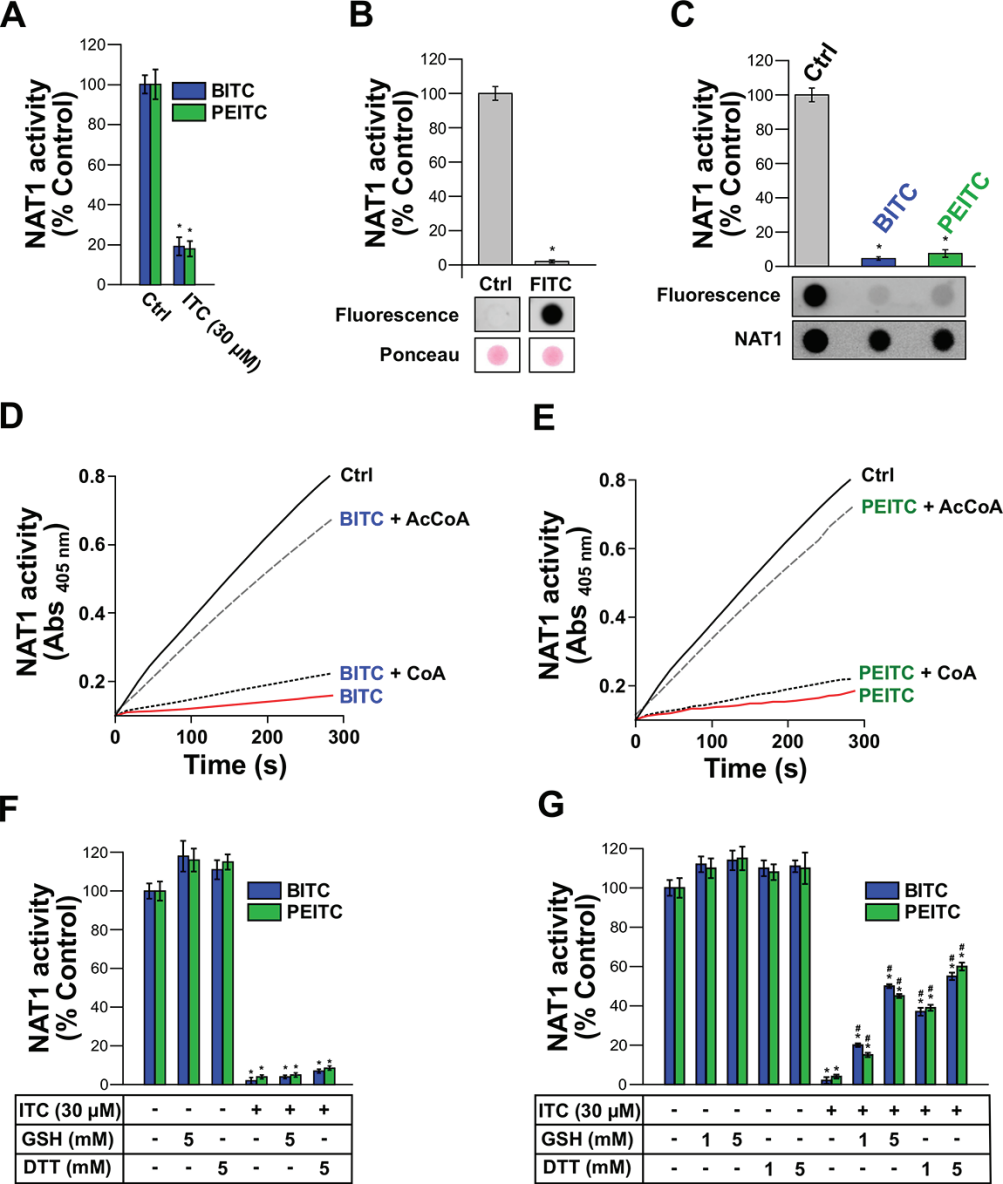
Irreversible inhibition of NAT1 by BITC and PEITC through covalent modification of NAT1 active-site catalytic cysteine (**A**) Effect of dialysis on the inhibition of NAT1 activity by BITC and PEITC. NAT1 enzyme (1 μM) was first incubated with BITC or PEITC (30 μM) for 30 min at 37°C. Samples were dialyzed overnight prior to residual NAT1 activity measurement. Error bars indicate S.D. values. Results are presented as percent control NAT1 activity. *p < 0.05 compared with NAT1 activity in the control. (**B**) ITC-coupled fluorescein (FITC) inhibits NAT1 activity and covalently binds to the enzyme. NAT1 enzyme (1 μM) was incubated with FITC (30 μM) for 30 min at 37°C and residual NAT1 activity measured. Error bars indicate S.D. values. Results are presented as percent control NAT1 activity. *p < 0.05 compared with NAT1 activity in the control. In parallel, samples were spotted on nitrocellulose membranes and FITC adducts determined by fluorescence. Membranes were also stained with Ponceau red. (**C**) Modification of NAT1 cysteines by BITC and PEITC. NAT1 (1 μM) enzyme was first incubated with BITC or PEITC (30 μM) and then incubated with 20 μM 5-(iodoacetamido)fluorescein (5-IAF) for 10 min at 37°C. Error bars indicate S.D. values. Results are presented as percent control NAT1 activity. *p < 0.05 compared with NAT1 activity in the controls. Samples were spotted on nitrocellulose membranes and 5-IAF labeling of NAT1 cysteine residues was determined by fluorescence. Membranes were also probed with an anti-NAT1 antibody. (**D, E**) Active-site protection assay using acetyl-coenzyme A (AcCoA) or coenzyme A (CoA). NAT1 enzyme (1 μM) was incubated with BITC (E) or PEITC. (**F**) (30 μM) in presence of an excess concentration (150 μM) of AcCoA or CoA. Progress curves for residual NAT1 activity (absorbance at 405 nm) are shown. (F) Effect of reduced glutathione (GSH) or dithiothreitol (DTT) on the inhibition of NAT1 activity by BITC and PEITC. NAT1 enzyme (1 μM) was first incubated with BITC or PEITC (30 μM) for 30 min at 37°C. Samples were further incubated for 30 min with reducing agents (5 mM DTT or 5 mM GSH) prior to residual NAT1 activity measurement. Error bars indicate S.D. values. Results are presented as percent control NAT1 activity. *p < 0.05 compared with NAT1 activity in the control. (**G**) GSH and DTT fail to fully protect NAT1 from inhibition by ITCs. NAT1 enzyme (1 μM) was preincubated with BITC or PEITC (30 μM) in the presence of high concentrations (> 160 times the concentration of BITC or PEITC) of GSH or DTT prior to residual activity measurement. Error bars indicate S.D. values. Results are presented as percent control NAT1 activity. *p < 0.05 compared with NAT1 activity in the controls. ^#^*p* < 0.05 compared with BITC or PEITC-inhibited NAT1.

Further molecular and kinetic analyses were carried out with human NAT1 in order to characterize the mechanisms underlying the inhibition of AA acetylation by BITC and PEITC. ITCs as electrophiles can react covalently with biological nucleophiles [19]. In particular, the covalent binding of ITCs to cysteines in certain target proteins (transcription factors, enzymes, cytokines, or cytoskeletal proteins) has been shown to play a key role in the biological effects of these phytochemicals [13, 17–19]. To test whether the inhibition of NAT1 by BITC and PEITC occurs through irreversible covalent modification of the enzyme, we first carried out dialysis experiments. As shown in Figure 2A, dialysis did not allow significant recovery of enzymatic activity of NAT1 previously exposed to BITC or PEITC, thus supporting that NAT1 inhibition by the two ITCs proceeds through an irreversible mechanism. Similar data were obtained with human NAT2 (Supplementary Figure 2A). To confirm that human NAT1 enzyme can be covalently modified by ITCs, we used fluorescein-coupled ITC (FITC) as a probe. We found that incubation of NAT1 with 30 μM FITC readily inhibited NAT1 with concomitant formation of fluorescein adducts on the protein (Figure 2B). No inhibition of NAT1 activity nor fluorescent labeling of the enzyme were obtained with fluorescein lacking the ITC moiety (data not shown). These experiments support that the inhibition of NAT1 by ITCs, such as BITC, PEITC or FITC occurs through irreversible covalent modification of the enzyme, which is in agreement with studies carried out with other proteins/enzymes targeted by ITCs [17–19]. Human NAT1 enzyme possesses 5 cysteine residues, one of which being present in the active site [20]. To investigate whether NAT1 cysteine residues are covalently modified, the enzyme was first inhibited by BITC or PEITC prior to labeling of free cysteine residues with fluorescein-conjugated iodoacetamide as described previously [21]. As shown in Figure 2C, incubation of the NAT1 with BITC and PEITC resulted in its inhibition and the concomitant modification of cysteine residues, as indicated by the disappearance of the fluorescein-conjugated iodoacetamide labeling. Thus, as observed for other proteins/enzymes targeted by ITCs, NAT1 cysteine residues are chemically-modified by BITC and PEITC [17–19]. Catalysis by NAT enzymes, including human NAT1, relies on the presence of a cysteine residue in the active site [20]. To test whether BITC and PEITC react with this cysteine residue, protection experiments in presence of AcCoA and CoA were carried out, as described previously [22]. This approach takes advantage of the catalytic mechanism of NAT enzymes which, in presence of AcCoA (but not in presence of CoA), involves the transient formation of a covalent acetyl-enzyme intermediate due to specific acetylation of the active-site cysteine [23, 24]. AcCoA protection experiments have been used to show that inhibition of NAT activity by chemicals is due (at least in part) to modification of the active site cysteine [22, 25]. As shown in Figure 2D and 2E, incubation of NAT1 with BITC or PEITC in the presence of AcCoA provided protection against ITCs-dependent inhibition. In contrast, CoA, which is unable to acetylate the active site cysteine of NAT1, did not afford protection. Taken together, the results presented above indicate that BITC and PEITC impair the acetylation of AAs by NAT1 through irreversible chemical modification of its active site cysteine. Similar data were obtained with human NAT2 (Supplementary Figure 2B and 2C). We further tested whether GSH and DTT, two chemicals that can reduce cysteine residues and react with ITCs, were able to reactivate BITC and PEITC-inhibited NAT1. As shown in Figure 2F, we found that GSH or DTT were unable to reactivate significantly inhibited NAT1, thus further supporting the irreversible nature of the BITC or PEITC reaction with the enzyme. Similar results were obtained with human NAT2 (Supplementary Figure 2D). GSH is a major cellular reducing agent which is known to be involved in the metabolic disposal of BITC and PEITC through uncatalyzed and catalyzed processes [26]. This occurs *via* reaction of the ITC moiety with the thiol group of GSH leading to the formation of GSH-dithiocarbamate conjugates [26]. We carried out experiments where NAT1 was incubated with BITC or PEITC in presence of an excess concentration of GSH or DTT over BITC or PEITC (33 and 166-fold excess). We found that these reducing agents only afforded partial protection against BITC and PEITC-dependent inhibition (close to 50% and 60% for 5 mM GSH and DTT, respectively) (Figure 2G). These data suggest that BITC or PEITC, even in the presence of high concentrations of GSH [21, 25], are more prone to react with the active site cysteine of NAT1 than with the thiol group of GSH. Similar results were obtained with human NAT2 (Supplementary Figure 2E).

**Figure 3:**
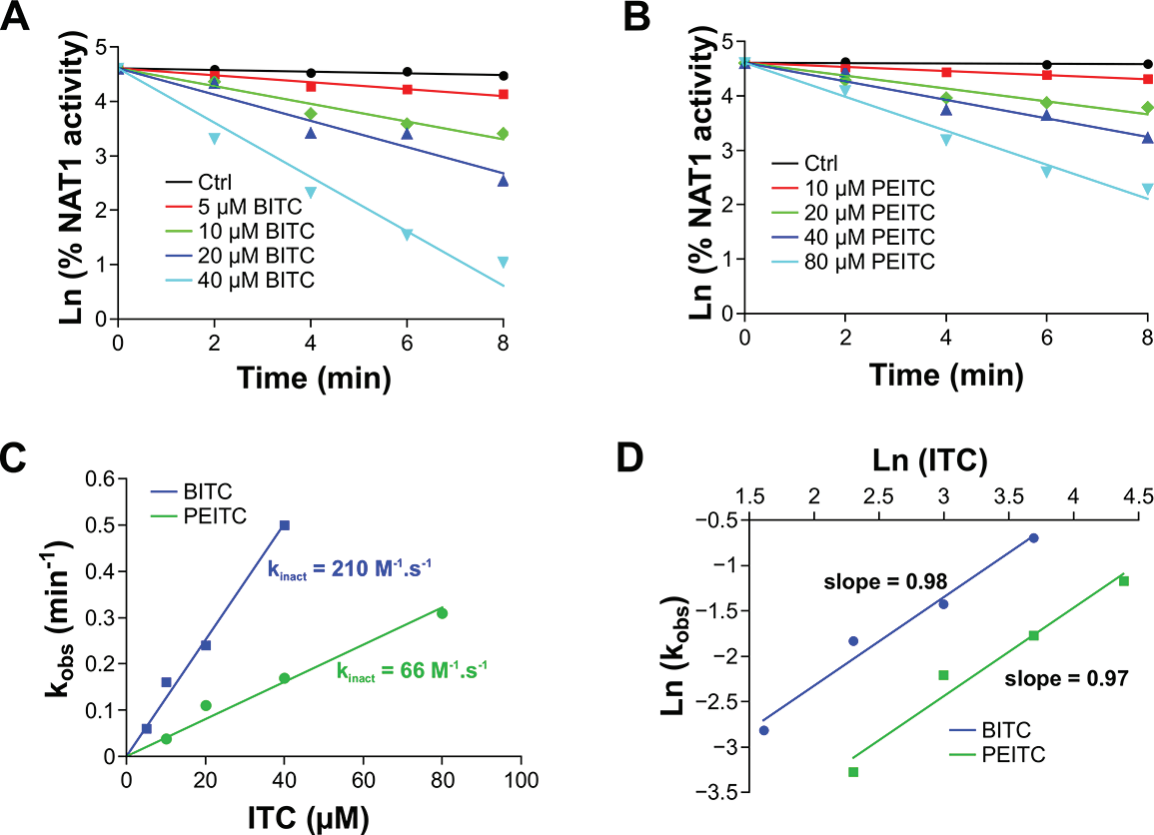
Kinetics analysis of ITCs-dependent inhibition of NAT1 NAT1 enzyme (1 μM) was incubated with increasing concentration of BITC or PEITC. Aliquots are taken every 2 min and residual NAT1 activity was measured. (**A, B**) Plots of the natural logarithm of the percentage residual activity *versus* time for each BITC (A) or PEITC (B) concentration. The apparent first-order inhibition constants (k_obs_) were calculated from linear regressions. (**C**) Determination of the second order inhibition constants (k_inact_). k_obs_ were plotted against BITC and PEITC concentrations and the k_inact_ were determined from the slopes. (**D**) Determination of the stoichiometry of the reaction of NAT1 with BITC or PEITC. Ln of k_obs_ were plotted as a function of ln ([BITC] or [PEITC]). The stoichiometries of the reactions for BITC or PEITC were calculated from the slopes.

To further characterize the reaction of BITC and PEITC with NAT1, kinetic analyses under pseudo-first order conditions were carried out. The enzyme was found to be inhibited in a time and dose-dependent manner by BITC and PEITC as shown in the semilogarithmic plots (Figure 3A and 3B). The plot of the apparent first-order inhibition constants (k_obs_) against BITC and PEITC concentrations fitted well to a line passing at the origin, indicating that inhibition of NAT1 by these two ITCs occurred through a single-step bimolecular process. The second-order rate constants for inhibition of NAT1 by BITC and PEITC were 210 and 66 M^−1^.s^−1^, respectively. These results are in agreement with previous studies indicating that BITC is more reactive than PEITC [17, 26]. In addition, the rate constants determined for the inhibition of NAT1 by BITC and PEITC are more than 100 times higher than those reported for the reaction of these ITCs with the thiol group of GSH (2 M^−1^.s^−1^ and 0.6 M^−1^.s^−1^, respectively) [26]. These differences may explain why GSH, even at concentrations well above those of BITC and PEITC did not provide full protection of the enzyme activity against ITC-dependent inhibition (Figure 2G). Analysis of the order of the reaction of BITC and PEITC with NAT1 further suggested that the inhibition of the enzyme occurred through a 1:1 stoichiometry which is in agreement with the AcCoA protection experiments reported above (Figure 2D and 2E). Altogether, our results suggest that the BITC and PEITC-dependent impairment of AAs acetylation occurs through the irreversible inhibition of NAT enzymes by a mechanism involving the chemical modification of the active site cysteine residue by the ITC moiety of the two phytochemicals.

**Figure 4:**
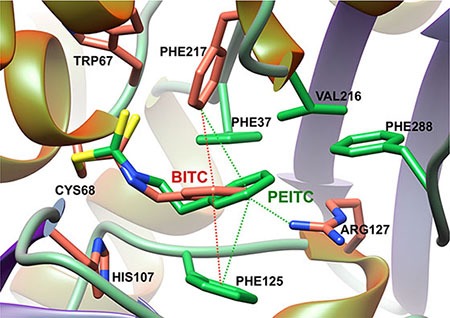
Model of BITC and PEITC bound to the catalytic cysteine of human NAT1 enzyme The binding modes of BITC and PEITC to NAT1 was initially obtained by covalent docking. Best conformation was minimized using Amber force field [38]. The figure was drawn by UCSF Chimera [39].

Docking approaches using the crystal structure of human NAT1 were consistent with these data. As shown in Figure 4, BITC and PEITC fitted well in the active site of the enzyme with the ITC moiety covalently bound to the catalytic cysteine and certain aromatic residues (Phe37, Phe125 and Phe217) making hydrophobic interactions with the aromatic ring of the arylalkyl side chains of BITC and PEITC (Figure 4). Interestingly, these aromatic amino acids are known to be involved in the binding of AcCoA and AA substrates in NAT active sites [20, 27, 28].

**Figure 5:**
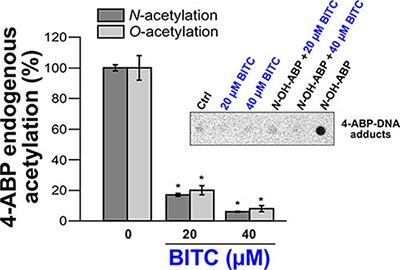
Effects of BITC on cellular NAT activities (*N*- and *O*-acetylation of 4-ABP) and on 4-ABP-DNA adducts For the determination of the endogenous *N*-acetylation activity of NAT1, MCF7 cells in culture dishes were first exposed to different concentrations of BITC for 30 min at 37°C in serum-free medium. Cell monolayers were then washed with medium and grown in the presence of 4-ABP (500 μM) for 4 h. *N*-acetyl-ABP was measured in cell culture medium by reverse phase high pressure liquid chromatography (RP-HPLC) as previously reported [5]. For the determination of the endogenous O-acetylation activity, after exposure to BITC, cells were lysed and 100 μg of cell extracts were incubated for 30 min at 37°C with 1 mM AcCoA, 100 μM *N*-OH-4-ABP and 1 mg/ml deoxyguanosine (dG). Quantification of deoxyguanosine-C8-ABP adducts was carried out by HPLC as previously reported [9]. Error bars indicate S.D. values. Results are presented as percent control endogenous NAT activity (*N*-or O-acetylation of 4-ABP/*N*-OH-4-ABP). *p < 0.05 compared with endogenous NAT activity (*N*-or O-acetylation of 4-ABP/*N*-OH-4-ABP) in the control. (*Inset*) 4-ABP-DNA adduct analysis. An immunodot blot assay was used to assess the formation of 4-ABP-DNA adducts in MCF7 cells exposed or not to BITC and *N*-OH-4-ABP as described previously [32]. MCF7 cells were first treated with BITC (20 μM or 40 μM) for 30 min in serum free medium. Cell monolayers were then washed with medium and grown in the presence of *N*-OH-4-ABP (250 μM) for 4 h. Cellular DNA was extracted, quantified and spotted on nitrocellulose membranes (500 ng/spot). 4-ABP-DNA adducts were detected using a monoclonal anti-4-ABP-DNA adduct antibody (4C11, Pierce Antibodies) as reported previously [32].

Cellular studies have shown that NAT1 and NAT2 activities are required for metabolic activation of 4-ABP. In particular, more 4-ABP DNA adducts, mutagenesis and cytotoxicity were detected in cells displaying high NAT1 and NAT2 activity [9, 10]. Therefore, in addition to the molecular and kinetic studies reported above, cellular approaches were carried out with BITC to further characterize the effects of ITCs on the enzymatic acetylation of AA carcinogens. To this end, the *N*- and *O*-acetylation of 4-ABP were measured in MCF7 cells exposed to BITC and 4-ABP (*N*-acetylation) or exposed to BITC and *N*-OH-4-ABP (*O*-acetylation) as described previously [5, 9]. The human breast cancer cell line MCF7 is known to have functional NAT1 and has been used previously to study the effects of ITCs on other XME [29, 30]. In addition, exposure to the 4-ABP has been associated to the etiology of breast cancer and 4-ABP-DNA adducts have been found in human breast cancer cells from smokers exposed to AA carcinogen [31]. As shown in Figure 5, short exposure of cultured cells to BITC significantly decreased the endogenous *N*- and *O*-acetylation of 4-ABP.

In parallel experiments, the levels of 4-ABP-DNA adducts in cells exposed or not to BITC and to *N*-OH-4-ABP were analyzed as described previously [32]. As expected exposure of cells to *N*-OH-4-ABP led to the formation of DNA adducts as reported previously [16] (Figure 5, Inset). Conversely, 4-ABP-DNA adducts were not detected in cells initially exposed to BITC which is consistent with the inhibition of the endogenous *O*-acetylation of *N*-OH-4 -ABP by BITC (Figure 5, Inset). These results are in agreement with previous reports showing that ITCs inhibit the formation of 4-ABP-DNA adducts in bladder cells and that these phytochemicals alter the metabolism of AA carcinogens and their carcinogenicity [15, 16]. Interestingly, it has been suggested that inhibition of hepatic NAT enzymes by cafestol and kahweol (two diterpene phytochemicals) and subsequent decreased bioactivation contribute to chemoprevention against cancers associated with heterocyclic AAs [33]. Activation of the Nrf2 signaling pathway by ITCs has been reported to be involved in the inhibition of 4-ABP-caused DNA damage, likely through the induction of GST that detoxifies 4-ABP [16].

Our experiments were carried out with ITC concentrations up to 40 μM. Several studies indicate that micromolar levels of ITC may be achieved *in vivo* [14]. In addition, it has been shown that ITCs, such as BITC, can accumulate in cells with intracellular concentrations 180 times higher than the initial extracellular ITC concentration [26]".

In conclusion, our data support that inhibition of NAT enzymes and subsequent alteration of the metabolic activation of AAs could be another key molecular mechanism by which ITCs, exert their chemoprotective effects towards AA carcinogens. Our study may provide a fondation for the development of NAT inhibitors and for the design of mechanism-based studies for the discovery of more efficacious ITC-related chemopreventive and therapeutic agents.

## MATERIALS AND METHODS

### Synthesis of *N*-OH-4-ABP

Melting points were measured in capillary tubes and are uncorrected. Proton (^1^H) NMR spectra were recorded on 300 MHz (QNP - ^13^C, ^31^P, ^19^F - probe or Dual ^13^C probe) and 500 MHz (BB0 - ATM probe or BBI - ATM probe) spectrometers. NMR experiments were carried out in deuterochloroform (CDCl_3_), chemical shifts (δ) are reported in parts per million (ppm) with reference to CDCl_3_ (^1^H: 7.26). The following abbreviations are used for the proton spectra multiplicities: s: singlet, d: doublet, t: triplet, q: quartet, m: multiplet, br: broad. Coupling constants (*J*) are reported in Hertz (Hz). High resolution mass spectra (HRMS) were obtained with an Electro-Spray Ionization Mass Spectrometer coupled with a Time of Flight analyzer (ESI-MS). Thin-layer chromatography was performed on silica gel 60 F_254_ on aluminum plates and visualized under UV and with TTC stain (1 g of 2,3,5-triphenyl tetrazolium chloride in 100 mL of water) for hydroxylamine (red spots). Flash chromatography was conducted on silica gel (40–63 μm) at medium pressure (300 mbar). All reagents were obtained from commercial suppliers unless otherwise stated. Where necessary, organic solvents were routinely dried and/or distilled prior to use and stored over molecular sieves under nitrogen.

*N*-hydroxylated 4-aminobiphenyl (*N*-OH-4-ABP) was synthesized from 4-nitrobiphenyl as described previously [34, 35]. A mixture of 1-chloro-4-nitrobenzene (1.0 g, 6.35 mmol), phenylboronic acid (1.10 g, 9.0 mmol), Pd(OAc)_2_ (45 mg, 0.2 mmol), 1,4-diazabicyclo [2.2.2] octane (DABCO, 45 mg, 0.4 mmol) and potassium carbonate (2.60 g, 6.0 mmol) in dimethylformamide (DMF, 40 mL) was stirred for 18 h at 110°C. The mixture was then cooled to room temperature, diluted with ethyl acetate, washed with water and extracted with ethyl acetate. The organic layers were dried with sodium sulfate and concentrated under vacuum. The crude residue was purified by flash column chromatography (heptane/ethyl acetate, 95/5) to afford 4-nitro-biphenyl (1.17 g, 92%). ^1^H NMR (300 MHz, CDCl_3_): *δ* (ppm) = 8.32 (d, *J =* 8.8 Hz, 2H, CH_Ar_), 7.76 (d, *J =* 8.8 Hz, 2H, CH_Ar_), 7.65 (d, *J =* 7.3 Hz, 2H, CH_Ar_), 7.54–7.51 (m, 2H, CH_Ar_), 7.49–7.46 (m, 1H, CH_Ar_). These data are in accordance with the literature [34, 35].

A suspension of 4-nitrobiphenyl (500 mg, 2.5 mmol) and ammonium chloride (230 mg, 4.3 mmol) in a mixture of methanol (10 mL) and water (1 mL) was preheated to 50°C. Zinc dust (750 mg, 11.5 mmol) was then added in small portions over a period of 2 min. The mixture was vigorously stirred and carefully monitored by TLC (TTC stain). After complete consumption of the starting material (ca. 10 min) the suspension was quickly filtered over celite and poured on ice. A precipitate formed that was extracted with diethyl ether, washed with brine, dried with sodium sulfate and concentrated under vacuum. The crude residue was washed with chloroform to give pure hydroxylaminobiphenyl (yellow solid). ^1^H NMR (500 MHz, CDCl_3_): *δ* (ppm) = 7.58 (d, *J =* 7.3 Hz, 2H, CH_Ar_), 7.55 (d, *J =* 8.3 Hz, 2H, CH_Ar_), 7.45–7.42 (m, 2H, CH_Ar_), 7.34–7.31 (m, 1H, CH_Ar_), 7.10 (d, *J =* 8.3 Hz, 2H, CH_Ar_); mp : 155°C; HRMS: m/z [M + H]^+^ calcd for C_12_H_12_NO: 186.0913 found 186.0898. These data are in accordance with the literature [34, 35].

### Production and purification of recombinant human NAT1

*Escherichia coli* BL21 (DE3) cells containing a pET28a-based plasmid were used to produce 6x-histidine-tagged human NAT1. Briefly, transformed bacterial cells were grown overnight at 16°C in the presence of 0.5 mM of isopropyl-1-thio-β-D-galactopyranoside. Cells were harvested by centrifugation and resuspended in lysis buffer (20 mM Tris HCl, pH 8, 300 mM NaCl, 0.1% Triton X-100, 1 mg/ml lysozyme and protease inhibitors (Sigma)). The lysate was subjected to sonication on ice and pelleted (12,000g; 30min). The supernatant was incubated with His-select nickel resin (Sigma) in the presence of 20 mM imidazole for 2 h at 4°C. Resin was poured into an empty column (Bio-rad) and washed with washing buffer (20 mM Tris-HCl pH 7.5, 150 mM NaCl and 35 mM imidazole). NAT1 was eluted in elution buffer (20 mM Tris-HCl pH 7.5, 150 mM NaCl and 300 mM imidazole). After reduction with 10 mM DTT (15 min on ice), purified NAT1 was applied to a PD-10 column (GE Healthcare) equilibrated with 20 mM Tris-HCl, pH 7.5, 150 mM NaCl. Protein concentration and purity were assessed by Bradford reagent (Bio-rad) and by SDS-PAGE. Proteins were kept at −80°C.

Purification of recombinant human NAT2 was carried out as described previously [25].

### Determination of the *N*-acetylation and *O*-acetylation activity of NAT1

BITC (Sigma), PEITC (Sigma) and NMPEA (Sigma) were diluted in DMSO at concentration of 100 mM. We tested the effect of ITCs on the activity of recombinant human NAT1 by incubating purified enzyme (1 μM final concentration) in phosphate-buffered saline (PBS) for 30 min at 37°C in absence or presence of different concentrations of ITCs. *N*-acetylation activity of purified human NAT1 in samples was determined spectrophotometrically in 96-wells plates using *p*-nitrophenylacetate (PNPA) as acetyl donor and *p*-aminobenzoic acid (PABA) as the arylamine substrate as previously described [6]. Briefly, ITC-treated or untreated samples were assayed in PBS containing PABA (500 μM final). Reactions (100 ml total volume) were started by addition of PNPA (200 μM final). The activity was determined at 37°C by monitoring for 15 min the increase in absorbance at 405 nm due to formation of *p*-nitrophenol using a microplate reader (BioTek).

*N*-acetylation activity of NAT2 enzyme was carried out with 2-aminofluorene (2-AF) as arylamine substrate.

*O*-acetylation activity of purified human NAT1 in samples was determined by reverse-phase HPLC using acetyl-coenzyme A (AcCoA) acetyl donor, *N*-OH-4-ABP as the *N*-hydroxylated arylamine substrate and deoxyguanosine [9]. Briefly, ITC-treated or untreated samples were assayed in PBS containing 100 μM *N*-OH-4-ABP, 1 mM AcCoA and 1 mg/ml deoxyguanosine at 37°C for 15 min. Reactions were stopped with the addition of 100 μL of water saturated ethyl acetate and centrifugated at 13, 000 g for 10 min. The organic phase was removed, evaporated to dryness and the residual was dissolved in 100 μL of 10% acetonitrile prior to HPLC quantification of deoxyguanosine-C8-ABP adducts as described previously [9].

All assays were performed in triplicate.

### Effects of fluorescein isothiocyanate (FITC) on purified NAT1 enzyme

NAT1 (1 μM) was incubated with or without 30 μM FITC in PBS for 30 min at 37°C. Residual NAT1 activity was measured using the PNPA assay as described above. Aliquots (corresponding to 1 mg NAT1) were also spotted on nitrocellulose membranes using a dot-blot apparatus (Bio-rad). To remove free FITC, membrane was washed with PBS and saturated 2 hours with PBS 5% milk. FITC adducts on spotted NAT1 were detected by fluorescence (Excitation: 494 nm, Emission: 512 nm) with a LAS-4000 apparatus (Fujifilm).

### Fluorescein-conjugated iodoacetamide labeling of NAT1 cysteines

NAT1 (1 μM) was incubated with 10 μM BITC or 10 μM PEITC in PBS for 30 min at 37°C. The reactions were quenched with DTT (5 μM) and further incubated with fluorescein-conjugated iodoacetamide (20 μM) for 10 min at 37°C (in the dark). Aliquots (corresponding to 1 mg NAT1) were also spotted on nitrocellulose membranes using a dot-blot apparatus (Bio-rad). Covalent modification of NAT1 cysteines by fluorescein-conjugated iodoacetamide were assessed by fluorescence (Excitation: 494 nm, Emission: 512 nm) with a LAS-4000 apparatus (Fujifilm). The membranes were further used for the immuno-detection of NAT1 using polyclonal anti-NAT1 antibody (166–180, Sigma).

### Effects of dialysis or reducing agents on ITC-inhibited NAT1

NAT1 (1 μM) were first incubated in PBS with 30 μM BITC or 30 μM PEITC for 30 min at 37°C. Samples were either dialysed overnight at 4°C against PBS or incubated 30 min with reducing agents (5 mM DTT or 5 mM GSH) at 37°C prior to residual NAT activity measurement using the PNPA assay. Experiments with NAT2 were carried out in similar manner excepted that 2-AF was used as arylamine substrate.

### Protection of NAT1 from ITC-dependent inhibition by reducing agents

NAT1 (1 μM) was incubated in PBS with 30 μM BITC or 30 μM PEITC in the presence of DTT or GSH (up to 5 mM) for 30 min at 37°C. Residual NAT activity was measured using the PNPA assay. Experiments with NAT2 were carried out in similar manner excepted that 2-AF was used as arylamine substrate.

### Effects of AcCoA and CoA on ITCs-dependent inhibition of NAT1

NAT1 (1 μM) was incubated in PBS with 30 μM BITC or 30 μM PEITC in the presence of 150 μM CoA or AcCoA for 30 min at 37°C. Residual NAT activity was measured using the PNPA assay. Experiments with NAT2 were carried out in similar manner excepted that 2-AF was used as arylamine substrate.

### Kinetic analysis of ITCs-dependent NAT1 inhibition

To determine the second-order rate inhibition constant of NAT1 by BITC and PEITC, the enzyme was preincubated with different concentrations of BITC and PEITC under pseudo first-order conditions. Briefly, NAT1 (1 μM final) was incubated with ITCs in PBS at 37°C. At different time intervals, aliquots were removed, diluted 10 times with PBS and assayed for residual NAT1 activity using the PNPA assay. The equation for the rate of inhibition of NAT1 by each ITC (BITC and PEITC) can be represented as −*d* [NAT1]/d*t* = *k*_i_.[NAT1]. [ITC] where [NAT1] is the concentration of enzyme and k_i_ the second-order inhibition rate constant. Provided that each ITC is present in substantial excess (pseudo-first order conditions), the apparent first order inhibition rate constants (k_obs_ = k_i_ × [ITC]) can be calculated for each ITC concentration from the slope of the natural log (ln) of percent residual activity plotted against time. The second-order rate constant (k_i_) was determined from the slope of k_obs_ plotted against ITC concentrations. Determination of the reaction order of the reaction of NAT1 enzyme with the BITC and PEITC was carried out by replotting the ln of k_obs_ *versus* the ln of ITC concentrations. Kinetic data were plotted and fitted using Qtiplot software (http://www.qtiplot.com/).

### Docking of BITC and PEITC in NAT1 active site

The binding modes of BITC and PEITC to NAT1 was initially obtained by covalent docking. The covalent bond between sulfur atom in the catalytic cysteine and the carbon atom in the thiocarbonyl group of BITC or PEITC was fixed by a strong gaussian potential with a weight of -100, and the width in gaussian function was set to 2.5, The conformation BITC or PEITC was searched by smina docking program [36, 37], with a customized score, which is contributed by Van der Waals interaction, hydrogen bond and a repulsion term for clashing. The top one conformation was then minimized by Ambertools [38]. The modified cysteine was assigned with GAFF force field and Amber99SB force field. The figure was drawn by UCSF Chimera [39].

### Determination of the NAT1 *N*- and O-acetyltransferase activities in MCF7 cells and exposure to BITC and AAs

MCF7 cells were grown in DMEM medium supplemented with 10% heat-inactivated fetal bovine serum (FBS) and 1 mM L-glutamine. MCF7 cells in six-well plates (2 × 10^6^ cells per well) were exposed to different concentrations of BITC for 30 min at 37°C in serum-free DMEM.

For the determination of the endogenous *N*-acetylation activity, cells exposed or not to BITC were washed with DMEM and grown in DMEM containing 500 μM 4-ABP. At different time points (2, 3, 4 h), 100 μL of medium were taken and mixed with 100 μL of HClO_4_. Mixtures were centrifuged 5 min at 10,000 g and the amount of acetylated-4-ABP was quantitated by reverse phase HPLC using a C18 column as previously described [5, 25].

For the determination of the endogenous *O*-acetylation activity, cell monolayers exposed to BITC were lysed (PBS containing 0.2% Triton X-100 and protease inhibitors) and centrifuged 10 min at 10,000 g. 100 μg of total cell extracts were incubated with 100 μL PBS containing 100 μM *N*-OH-4-ABP, 1 mM AcCoA and 1 mg/ml deoxyguanosine at 37°c for 15 min. Formation of deoxyguanosine-C8-ABP adducts was measured by HPLC as described above.

### Determination of 4-ABP-DNA adducts

We used an immunodot blot assay to assess the formation of 4-ABP adducts on cellular DNA from MCF7 cells. Briefly, MCF7 cell monolayers in six well plate (2 × 10^6^ cells per well) were first exposed to BITC in free-serum DMEM for 30 min at 37°C. After a wash with DMEM, cells were grown in DMEM containing 250 μM *N*-OH-4-ABP for 4 h. Cellular DNA was extracted using a Trizol kit (Euromedex) and quantitated with a NanoDrop 2000 apparatus. Heat-denaturated DNA (500 mg) was dot blotted onto a nitrocellulose membrane (Amersham Protran 0.2 NC) using the dot blot apparatus (Bio-rad). 4-ABP-DNA adducts on spotted DNA samples were detected using a monoclonal anti-4-ABP-DNA adduct antibody (4C11, Pierce Antibodies) as reported previously [32].

### Statistical analysis

Data are means ± S.D. of three independent experiments. One-way ANOVA was performed and followed by Bonferroni correction using Statview 5.0 (SAS Institute Inc., USA).

## SUPPLEMENTARY MATERIALS FIGURES


