# Supplementary material for: Identification of cancer chemopreventive isothiocyanates as direct inhibitors of the arylamine N-acetyltransferase-dependent acetylation and bioactivation of aromatic amine carcinogens
Source: Oncotarget. 2016 Jan 30;7(8):8688–99. doi: 10.18632/oncotarget.7086 (PMC4890997; doi:10.18632/oncotarget.7086)
Supplement: Supplementary file 1 [file oncotarget-07-8688-s001.pdf]

Identification of cancer chemopreventive isothiocyanates as direct inhibitors of the arylamine *N*-acetyltransferase-dependent acetylation and bioactivation of aromatic amine carcinogens

Supplementary Materials

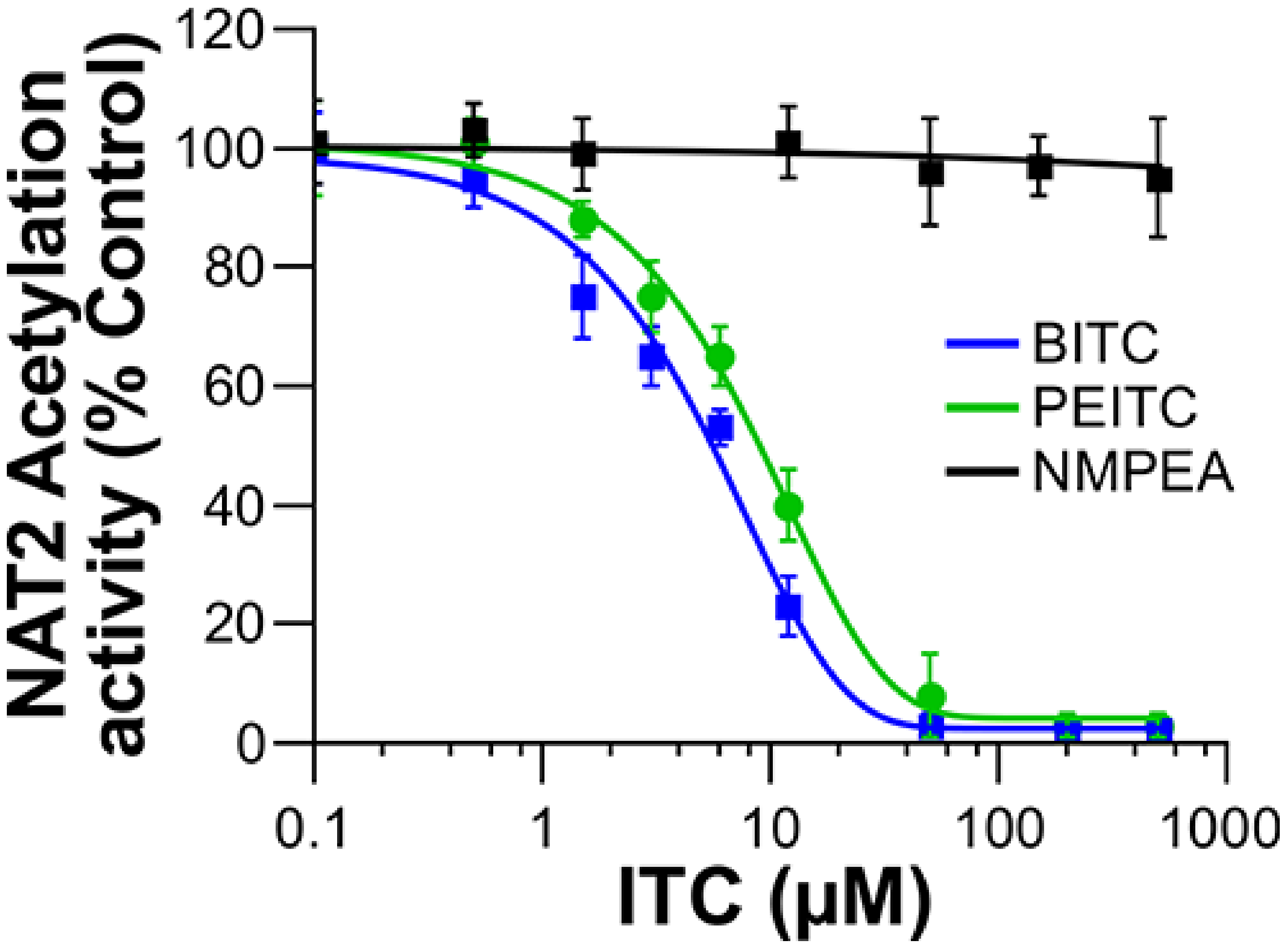

Supplementary Figure S1: Dose-dependent inhibition of NAT2 activity by the aralkyl isothiocyanates benzyl isothiocyanate (BITC) and phenetyl isothiocyanate (PEITC). Residual NAT2 N-acetylation was measured using 2-aminofluorene (2-AF) as AA substrates. Error bars indicate S.D. values. Results are presented as percent control NAT2 activity.

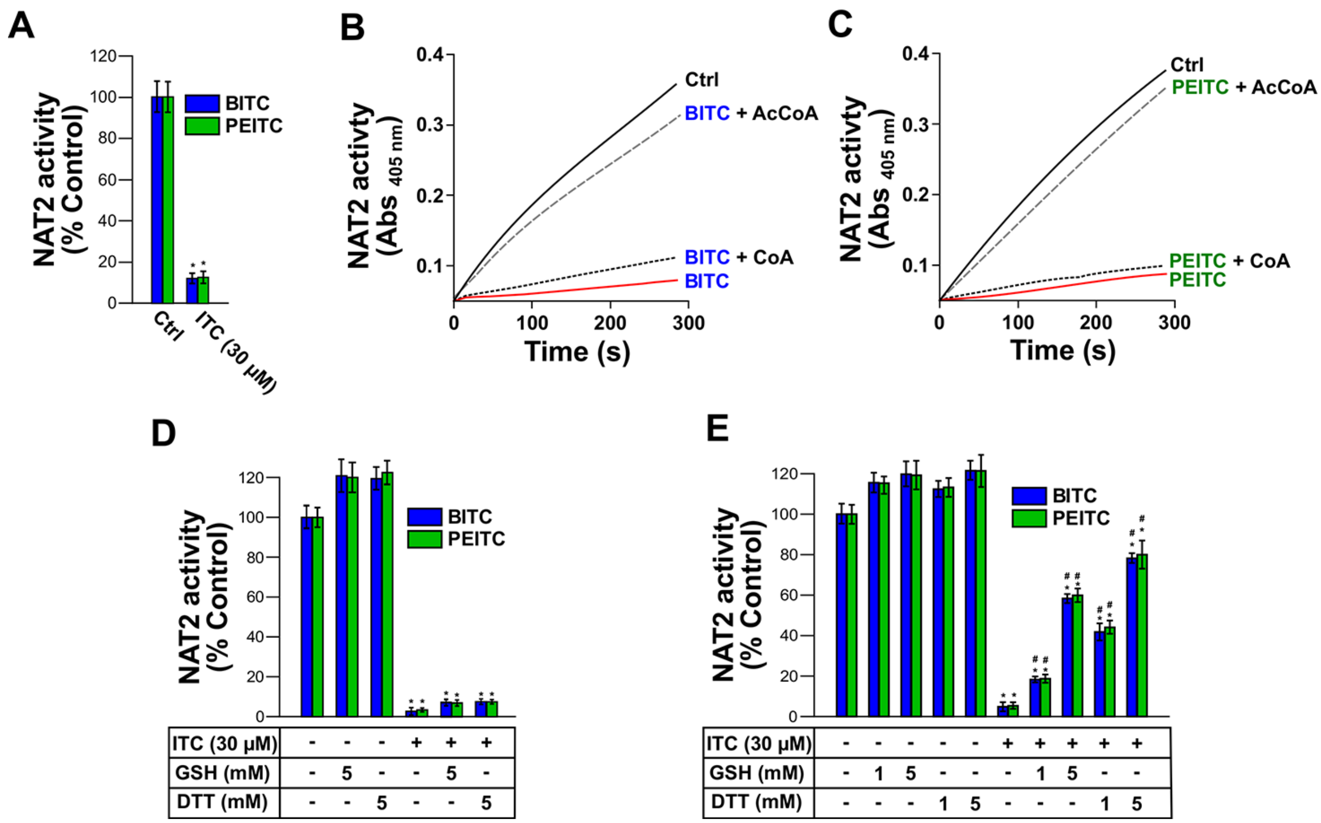

Supplementary Figure S2: Irreversible inhibition of NAT2 by BITC and PEITC through modification of NAT2 active-site. (**A**) Effect of dialysis on the inhibition of NAT2 activity by BITC and PEITC. NAT2 enzyme (1 µM) was first incubated with BITC or PEITC (30 µM) for 30 min at 37°C. Samples were dialyzed overnight prior to residual NAT2 activity measurement. Error bars indicate S.D. values. Results are presented as percent control NAT2 activity. *$p < 0.05$ compared with NAT2 activity in the control. (**B**, **C**) Active-site protection assay using acetyl-Coenzyme A (AcCoA) or Coenzyme A (CoA). NAT2 enzyme (1 µM) was incubated with BITC (B) or PEITC (C) (30 µM) in presence of an excess concentration (150 µM) of AcCoA or CoA. Progress curves for residual NAT2 activity (absorbance at 405 nm) are shown. (**D**) Effect of reduced glutathione (GSH) or dithiothreitol (DTT) on the inhibition of NAT2 activity by BITC and PEITC. NAT2 enzyme (1 µM) was first incubated with BITC or PEITC (30 µM) for 30 min at 37°C. Samples were further incubated for 30 min with reducing agents (5 mM DTT or 5 mM GSH) prior to residual NAT2 activity measurement. Error bars indicate S.D. values. Results are presented as percent control NAT2 activity. *$p < 0.05$ compared with NAT2 activity in the control. (**E**) GSH and DTT fail to fully protect NAT2 from inhibition by ITCs. NAT2 enzyme (1 µM) was preincubated with BITC or PEITC (30 µM) in the presence of high concentrations (> 160 times the concentration of BITC or PEITC) of GSH or DTT prior to residual activity measurement. Error bars indicate S.D. values. Results are presented as percent control NAT2 activity. *$p < 0.05$ compared with NAT2 activity in the controls.$^{\#}p < 0.05$ compared with BITC or PEITC-inhibited NAT2.
